# Early Changes in Hippocampal Neurogenesis in Transgenic Mouse Models for Alzheimer’s Disease

**DOI:** 10.1007/s12035-016-0018-9

**Published:** 2016-08-20

**Authors:** M. S. Unger, J. Marschallinger, J. Kaindl, C. Höfling, S. Rossner, Michael T. Heneka, A. Van der Linden, Ludwig Aigner

**Affiliations:** 1 Institute of Molecular Regenerative Medicine, Paracelsus Medical University, Strubergasse 21, 5020 Salzburg, Austria; 2 Spinal Cord Injury and Tissue Regeneration Center Salzburg (SCI-TReCS), Paracelsus Medical University, Salzburg, Austria; 3 Paul Flechsig Institute for Brain Research, University of Leipzig, Leipzig, Germany; 4 Clinical Neuroscience, Department of Neurology, University of Bonn, Bonn, Germany; 5 Department of Biomedical Sciences, University of Antwerp, Antwerp, Belgium

**Keywords:** Neurogenesis, Neural stem- and progenitors, Alzheimer’s disease

## Abstract

Alzheimer’s disease (AD) is the most prevalent neurodegenerative disease in the Western world and is characterized by a progressive loss of cognitive functions leading to dementia. One major histopathological hallmark of AD is the formation of amyloid-beta plaques, which is reproduced in numerous transgenic animal models overexpressing pathogenic forms of amyloid precursor protein (APP). In human AD and in transgenic amyloid plaque mouse models, several studies report altered rates of adult neurogenesis, i.e. the formation of new neurons from neural stem and progenitor cells, and impaired neurogenesis has also been attributed to contribute to the cognitive decline in AD. So far, changes in neurogenesis have largely been considered to be a consequence of the plaque pathology. Therefore, possible alterations in neurogenesis before plaque formation or in prodromal AD have been largely ignored. Here, we analysed adult hippocampal neurogenesis in amyloidogenic mouse models of AD at different points before and during plaque progression. We found prominent alterations of hippocampal neurogenesis before plaque formation. Survival of newly generated cells and the production of new neurons were already compromised at this stage. Moreover and surprisingly, proliferation of doublecortin (DCX) expressing neuroblasts was significantly and specifically elevated during the pre-plaque stage in the APP-PS1 model, while the Nestin-expressing stem cell population was unaffected. In summary, changes in neurogenesis are evident already before plaque deposition and might contribute to well-known early hippocampal dysfunctions in prodromal AD such as hippocampal overactivity.

## Introduction

Alzheimer’s disease (AD) is an age-related neurodegenerative disease that leads to a progressive loss of cognitive functions [reviewed in 1, 2]. The most prominent histopathological hallmarks contributing to the cognitive decline are the formation of amyloid-beta plaques and neurofibrillary tangles [reviewed in 3, 4]. Whereas the mechanism of amyloid-beta plaque formation is still not fully understood, it is widely accepted that low molecular weight amyloid-beta aggregates such as oligomers act as toxic agents harming synapses and neurons, leading to altered synaptic function, neuronal loss, massive neuroinflammation, and finally contribute to dementia [reviewed in 5, 6]. Based on this, a number of transgenic AD animal models have been generated to simulate the amyloid-beta plaque burden found in humans and to study familial Alzheimer’s disease forms (FAD) with their human relevant genetic predispositions [7–9]. Two widely used AD mouse models are the single transgenic Tg2576 expressing the human APP with the Swedish mutation (Tg(HuAPP695. K670N- M671L)2576) [10] and the double transgenic APP Swedish PS1 dE9 mouse model, in which mice are expressing APP and PS1 mutations associated with early-onset AD forms, i.e. a chimeric mouse/human amyloid precursor protein (Mo/HuAPP695swe) and a mutant human presenilin 1 (PS1-dE9) both directed to CNS neurons under the prion protein promoter [11, 12]. Whereas the Tg2576 mouse model is a slow progressive mouse model developing amyloid-beta plaques and deficits in learning and memory at 9–13 months of age [10, 13], in the APP Swedish PS1 dE9 mouse model, amyloid-beta plaques start to form between an age of 3–4 and 9 months in the cortex and hippocampus [14–17]. Cognitive deficits in spatial working memory were observed starting already with 3–5 months of age in the double transgenic mouse model [reviewed in 18].

Adult hippocampal neurogenesis has been explored in the context of AD [reviewed in 19], as alterations in neurogenesis might contribute to cognitive dysfunctions in dementias, and as stimulation of hippocampal neurogenesis might counteract the cognitive decline [reviewed in 7, 20, 21]. In the adult rodent and human hippocampus, new neurons are generated and integrated into the hippocampal circuitry from so-called neural stem- and progenitor cells (NPCs) that reside in the subgranular cell layer (SGCL) of the dentate gyrus [22, 23, 24, 25, reviewed in 26]. Importantly, hippocampal neurogenesis is crucially involved in learning and memory [27, 28, reviewed in 29].

In AD and in animal models of AD, neurogenesis has been analysed, however with somewhat controversial results [30–33]. Nevertheless, the vast majority of studies report about decreased neurogenesis in AD mouse models often coinciding with amyloid-beta plaque pathology [reviewed in 21, 34]. For example, adult hippocampal neurogenesis is decreased in mice overexpressing the human APP with the Swedish mutation [31] but in mouse models harbouring the APP and the PS1 mutated genes decreased levels of neurogenesis [17] and increased levels of neurogenesis were reported in mice developing memory impairments and progressive plaque pathology [35]. In contrast, neurogenesis was found to be increased in the APP model expressing the Swedish and Indiana mutation [30]. In human AD patients, the number of cells expressing young immature neuronal markers was elevated compared to age-matched healthy controls [36]. The differences described might rely on the different genotypes depending on the analysed AD mouse models, on the different methods used to asses neurogenesis, and of course, also on the different stages of progression of the disease or of the pathology [reviewed in 20, 21]. However, very little is known about differences in neurogenesis at the beginning of the disease or before the onset of amyloid-plaque formation.

As there is increasing awareness for the importance of the pre-symptomatic phase in neurodegenerative diseases in the context of early diagnosis and of pathogenesis [reviewed in 37, 38], we investigated the temporal pattern of amyloid-beta plaque deposition in the hippocampus and analysed corresponding levels of adult hippocampal neurogenesis from very early prodromal to severe stages of amyloid-beta plaque formation in the APP Swedish PS1 dE9 mouse model and in the slower progressing Tg2576 AD mouse model.

## Experimental Procedures

### Animals

We used female APP Swedish PS1 dE9 mice [reviewed in 12] expressing a chimeric mouse/human mutant amyloid precursor protein (Mo/HuAPP695swe) and a mutant human presenilin 1 (PS1-dE9) both directed to CNS neurons under the prion protein promoter (Jackson Laboratory, https://www.jax.org/strain/005864). We analysed mice at 3, 10, and 13 months of age. Age-matched non-transgenics derived from the APP Swedish PS1 dE9 breeding were used as control animals (*n* = 6/group). Mice were housed at the University Hospital Bonn—Clinical Neuroscience in groups under standard conditions at a temperature of 22 °C and a 12 h light/dark cycle with ad libitum access to standard food and water. Animal care and handling were performed according to the Declaration of Helsinki and approved by local ethical committees. Transgenic female Tg2576 mice overexpressing human APP Swedish and wild type littermates at postnatal ages of 3 and 5 months (*n* = 6/group) were housed at the Medizinisch-Experimentelles Zentrum, University of Leipzig, at 12 h light/12 h dark cycles with standard food and water ad libitum. The use of experimental animals with Tg2576 was approved by the Landesdirektion Sachsen (licence 06/15).

### Bromodeoxyuridine Injection

For histological analysis of cell survival and fate, APP Swedish PS1 dE9 mice received intraperitoneal injections of 10 mg/ml of the thymidine analogue bromodeoxyuridine (BrdU) (Sigma) at 50 mg/kg of body weight dissolved in 0.9 % NaCl solution daily on 5 consecutive days 4 weeks before perfusion.

### Perfusion and Tissue Processing

APP Swedish PS1 dE9 and Tg2576 animals were anaesthetised and transcardially perfused for immunohistochemistry as previously described [39, 40]. Following perfusion, the brains were extracted and postfixed in 4 % paraformaldehyde (PFA), sodium phosphate buffer solution (PBS, 0.1 M; pH = 7.4) over night at 4 °C. The brains were cryoprotected and transferred into 30 % sucrose, 0.1 M sodium phosphate buffer solution (pH = 7.4). Sagittal sections of 40 μm (APP Swedish PS1 dE9) and coronal sections of 30 μm (Tg2576) were cut on dry ice, using a sliding microtome.

### Immunohistochemistry

Fluorescence Immunohistochemistry was performed on free-floating sections. Following washes of PBS (0.1 M, pH = 7.4), antigen retrieval was performed by steaming the sections for 15–20 min in citrate buffer (pH = 6.0, Sigma). For BrdU and PCNA labelling, the following steps were performed: the slices were incubated for 1 h at 65 °C in 50 % formamide (Merck) diluted in saline sodium citrate (SSC; 0.3 M NaCl, 30 mM Na_3_Ci, pH = 7.0, Sigma), washed in SSC, incubated in 2 M HCl (Merck) for 30 min at 37 °C, followed by incubation in 0.1 M borate buffer (pH = 8.5) for 10 min at room temperature (RT). Brain sections were washed in PBS and afterwards blocked for 1 h in FSGB + T (1 % BSA, 0.2 % fish skin gelatine and 0.1 % Triton X-100 in PBS, all from Sigma). Incubation with primary antibodies was done over night at room temperature (RT). The following primary antibodies were used: rabbit anti-doublecortin (1:300, Cell Signaling), goat anti-PCNA (1:300, Santa Cruz Biotechnology), mouse anti-NeuN (1:500, Millipore), mouse anti-Nestin (1:300, Abcam), rabbit anti-Iba1 (1:300, Wako), rat anti-BrdU (1:500, AbD Serotec), rabbit anti-Olig2 (1:200, Millipore), guinea pig anti-GFAP (1:500, Progen Biotechnik), mouse anti-Abeta (1:1000, Covance).

On the next day, sections were extensively washed in PBS and incubated for 3 h at RT in secondary antibodies diluted in FSGB + T. The following secondary antibodies were used: donkey anti-rabbit Alexa Fluor 568, donkey anti-mouse Alexa Fluor 488, anti-mouse Alexa Fluor 568, donkey anti-mouse Alexa Fluor 647, donkey anti-rat Alexa Fluor 488 (all Invitrogen), donkey anti-goat Alexa Fluor 647 (Jackson Immuno Research), donkey anti-guinea pig Alexa Fluor 647 (Dianova), all 1:1000.

Nucleus counterstaining was performed with 4′.6′-diamidino-2-phenylindole dihydrochloride hydrate (DAPI 1 mg/mL, 1:2000, Sigma). For qualitative amyloid-beta plaque staining, Thioflavin S (1 mg/mL, 1:625, Sigma) was added to the secondary antibody solution. Sections were washed in PBS after incubation with secondary antibodies and tissue from old mice (>9 months) was additionally treated with 0.2 % Sudan Black (Sigma) in 70 % EtOH for 1–2 min to reduce the autofluorescence of lipofuscin in old tissue. After this treatment, the sections were extensively washed in PBS and mounted onto microscope glass slides (Superfrost Plus, Thermo Scientific). Brain sections were cover slipped semi dry in ProLong Gold Antifade Mountant (Life technologies) or Fluorescence Mounting Medium (Dako).

### Microscopy, Visualization and Image Processing

For microscopic analysis of the plaque area and analysis of adult hippocampal neurogenesis, Confocal Laser Scanning Microscopy was performed using a LSM 700 or LSM 710 from Zeiss. Representative images were obtained by orthogonal projections (maximum intensity) and depicted in respective false colours using Zen 2012 blue edition software. For quantification of the GCL volume, images of the dentate gyrus were taken with an Olympus fluorescence microscope (Olympus IX81).

### Quantitative Analysis of Immunohistological Data

#### Volume Analysis of Dentate Gyrus Granular Cell Layer

To estimate changes in the volume of the dentate gyrus granular cell layer (GCL) between WT and APP Swedish PS1 dE9 animals, the DAPI and Thioflavin S stained area of the dentate gyrus GCL from one tenth of the brain hemisphere was measured for each animal using ImageJ (1.47v). The GCL volume for one hemisphere was calculated by multiplication with 10 (multiplication factor for whole brain hemisphere) and 40 (section thickness) for every animal (*n* = 3/group). Statistical analysis was performed with GraphPad Prism 6.

#### Quantification of Amyloid-Beta Plaque Areas

Five plaques in the hippocampus and 5 plaques in the cortex were analysed in three 10- and 13-month old APP Swedish PS1 dE9 animals and the area of the amyloid-beta stained plaque was measured using the LSM Image Browser software (Version 4.2.0.121, Zeiss). Statistical analysis was done with GraphPad Prism 6.

#### Quantification of Adult Hippocampal Neurogenesis

For the APP Swedish PS1 dE9 mouse model, all qualitative and quantitative analysis were performed in the dorsal dentate gyrus of the hippocampus of 40 μm sagittal sections. For quantification, every tenth section (400 μm intervals) of one brain hemisphere was selected from each animal (*n* = 6/group), and per animal four randomly selected visual fields of the DGs were analysed. Antibody-positive cell types were counted and quantified in the GCL and SGCL. The number of proliferating cells (PCNA^+^), neuroblasts (DCX^+^), stem cells (Nestin^+^), microglia (Iba1^+^) and double positive cells were assessed. Additionally, the number of BrdU^+^ cells and the number of newly formed cells such as neuroblasts (BrdU^+^/DCX^+^), neurons (BrdU^+^/NeuN^+^), oligodendrocytes (BrdU^+^/Olig2) and astrocytes (BrdU^+^/GFAP^+^) were quantified. The corresponding tissue area, i.e. GCL and SGCL, was measured and multiplied by 40 to obtain the tissue volume represented in cubic micrometre. To assess cell densities, the total number of counted cells per animal was divided by the dentate gyrus volume and represented as cells/cubic millimetre.

For analysis of hippocampal neurogenesis in the Tg2576 animal model, six randomly picked visual fields of DGs per animal of 30 μm coronal sections (−1.46 to −3.16 mm from Bregma) were selected. The number of proliferating cells (PCNA^+^), neuroblasts (DCX^+^) and double positive cells (PCNA^+^/DCX^+^) was counted in the GCL and SGCL of the dentate gyrus. The corresponding tissue area of GCL and SGCL was measured and multiplied by 30 to obtain the tissue volume represented in cubic micrometre. To assess cell densities, the total number of counted cells was divided by the dentate gyrus volume and represented as cells/cubic millimetre.

All analysis were performed using the ImageJ (1.47v) software. Statistical analysis was done with GraphPad Prism 6.

#### Statistical Analysis

Statistical analysis was performed using the GraphPad Prism 6 (GraphPad Software). Data were tested for normality using the D’Agostino-Pearson omnibus normality test and outliers were identified by Grubbs’ test. Values between two groups were compared by the two-tailed unpaired Student’s *t* test for normally distributed data. Comparison of multiple groups was done by two-way analysis of variance (ANOVA) with Tukey multiple comparison.

*P* values of *p* < 0.0001 (****) and *p* < 0.001 (***) were considered most significant, *p* < 0.01 (**) highly significant, and *p* < 0.05 (*) significant. Values were expressed as means ± standard deviation (SD).

## Results

### Amyloid-Beta Plaques Increase in Size and Disrupt the GCL of the Dentate Gyrus in APP Swedish PS1 dE9 Mice

We first aimed to determine the onset and the progression of amyloid-beta plaque formation in the hippocampus of APP Swedish PS1 dE9 mice, herein abbreviated APP-PS1 mice. In this transgenic mouse model, Thioflavin S positive amyloid-beta plaques are described to form at 3 months [17] or at 4 months of age in hippocampal and cortical regions [14]. As the main scope of the present study was to focus on hippocampal neurogenesis in relation to plaque progression, we first analysed amyloid-beta plaque deposition in the hippocampus by Thioflavin S staining at different ages of the APP-PS1 animals.

**Fig. 1:**
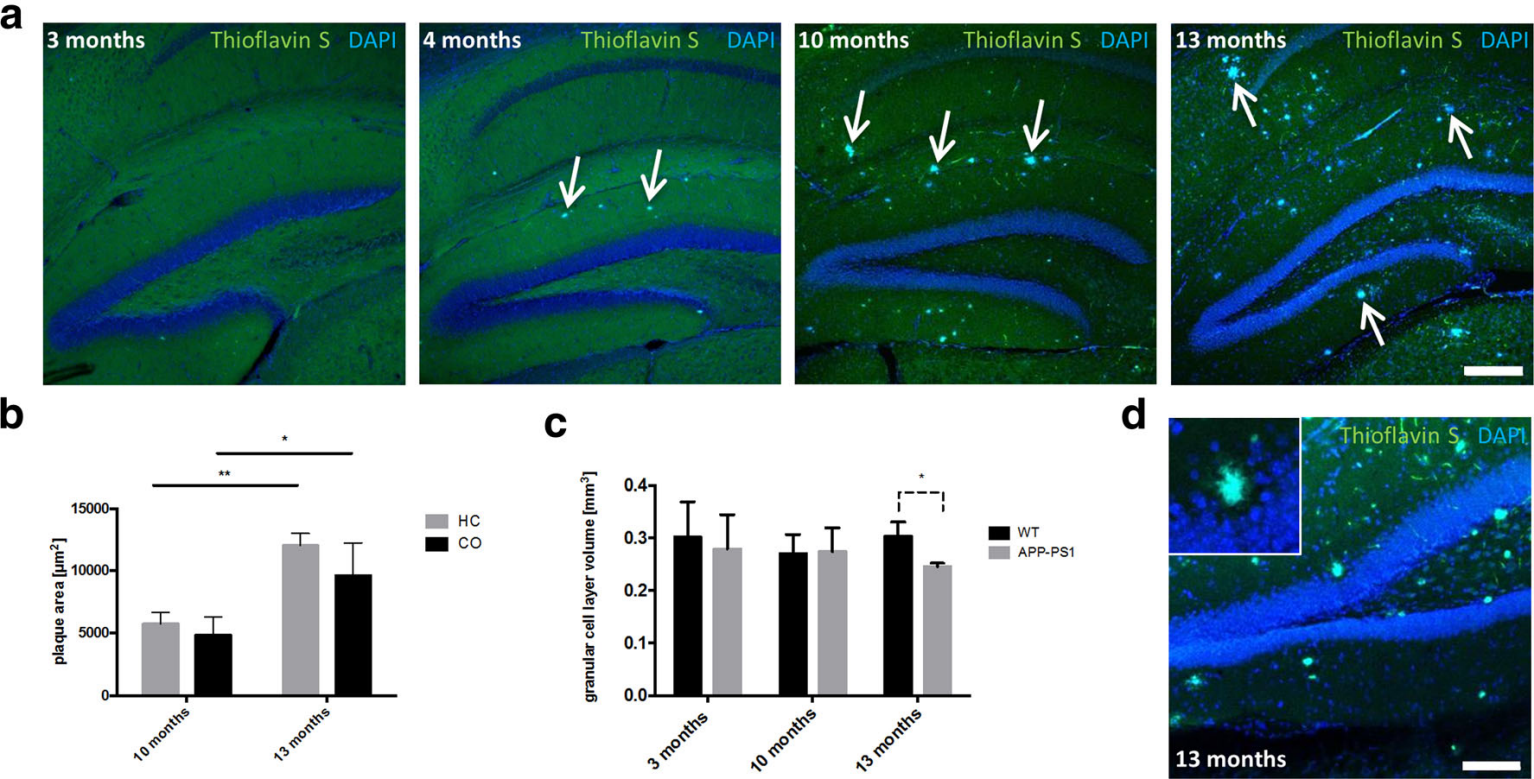
Development of amyloid-beta plaques in the hippocampus of APP-PS1 mice at different time points of AD progression. **a** Amyloid-beta plaques are first observed in the hippocampus of APP-PS1 mice at an age of 4 months and increased in number over time. **b** The area of Abeta antibody immunoreactive plaques significantly increased in the hippocampus of 13-month-old compared to 10-month-old AD animals. The same increase in amyloid-beta plaque load was observed in the cortex. **c** Analysis of the GCL volume in the dorsal dentate gyrus of the hippocampus revealed significantly decreased GCL volume specifically in 13-month-old APP-PS1 mice compared to WT (unpaired Student’s *t* test, *p* = 0,0213). **d** With progression of Abeta pathology, amyloid-beta plaques appear in the GCL of the dentate gyrus perforating the healthy tissue structure. *Arrows* show amyloid-beta plaques stained with Thioflavin S. DAPI was used to stain cell nuclei. Two-way ANOVA with Tukey’s multiple comparisons test (**b**) and unpaired Student’s *t* test (**c**) was performed (*n* = 3/group). Scale 200 μm (**a**), 100 μm (**d**)

Thioflavin S positive amyloid-beta deposits were detected in the hippocampus of 4 months old APP-PS1 mice, whereas 3-month-old transgenic animals were mostly devoid of any plaque deposits (Fig. 1a). In the Tg2576 AD mouse model, brains were free of amyloid-beta plaques at 3 months of age, sporadically, we observed small Thioflavin S positive plaque deposits at 5 months, while prominent plaque load was evident from 8 months on (data not shown). Amyloid-beta plaque load increased with age and was more prominent in the 10-month and even more in the 13-month-old APP-PS1 animals (Fig. 1a). Quantitative analysis revealed that the size of individual plaques, both in the hippocampus and in the cortex, still increased between 10 and 13 months of age (Fig. 1b). Brains of WT animals, regardless of age, were completely devoid of any amyloid-beta plaque depositions (data not shown).

Since hippocampus atrophy is a hallmark of AD, we evaluated possible volume changes of the dentate gyrus GCL in the hippocampus of the APP-PS1 mice. Whereas the 3- and 10-month-old animals did not display any differences in the GCL volume, the 13-month-old AD animals showed a significantly decreased GCL volume compared to age-matched WT controls (Fig. 1c). Interestingly, with progression of plaque pathology, we observed amyloid-beta plaques being located in the GCL of the hippocampus perforating the dentate gyrus structure (Fig. 1d). Thus, besides a general atrophy of the GCL, amyloid-beta plaque formation in the GCL and associated loss of neurons might contribute to GCL volume changes at late stages of AD pathology.

Taken together, small Thioflavin S positive amyloid-beta plaques were observed to form at 4 months of age in the hippocampus of APP-PS1 mice. With increased age, amyloid-beta plaque load was significantly increased in the hippocampus as well as in the cortex and amyloid-beta deposits were found to perforate the GCL structure most likely contributing to neurodegenerative events along pathology.

### Hyperproliferation of Immature DCX Positive Neuroblasts at Pre-plaque Stages in APP-PS1 and Tg2576 Mice

Quantitative analysis of adult hippocampal neurogenesis in the GCL and SGCL of the dentate gyrus in the APP-PS1 mice and in Tg2576 mice was performed by immunohistochemistry. The rate of neurogenesis was determined by evaluating the numbers of proliferating cells (PCNA^+^), neuroblasts (DCX^+^) and proliferating neuroblasts (PCNA^+^/DCX^+^) at different time points. Furthermore, the stem cell pool was analysed by quantifying the numbers of Nestin^+^ cells.

**Fig. 2:**
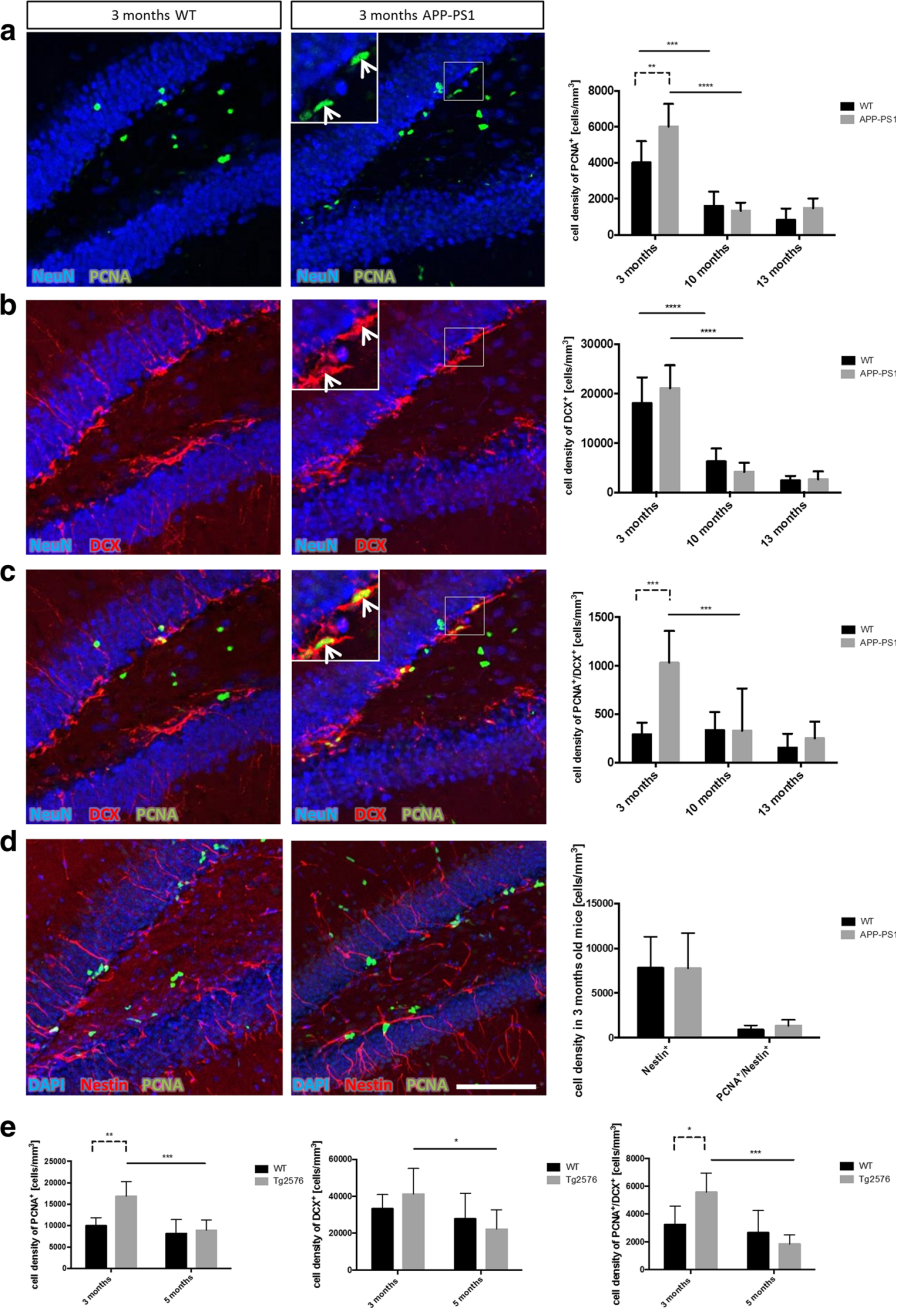
Hyperproliferation of immature DCX positive neuroblasts at early stages of Abeta pathology in transgenic animal models. Quantitative analysis of adult hippocampal neurogenesis in the GCL and SGCL of the dentate gyrus in APP-PS1 and Tg2576 mice was performed by immunohistochemical staining for proliferating cells (PCNA^+^), neuroblasts (DCX^+^) and proliferating neuroblasts (PCNA^+^/DCX^+^) at different time points of Abeta pathology progression. **a** While PCNA^+^ cells decrease with age from 3 to 10 months in both WT and TG, the 3-month-old APP-PS1 animals showed a significant increase in the number of PCNA^+^ cells compared to WT. **b** The number of DCX^+^ cells was not changed between WT and APP-PS1 mice in any age group; however, both showed decreased DCX^+^ cell numbers with increased age. **c** The 3-month-old APP-PS1 mice had significantly elevated numbers of PCNA^+^/DCX^+^ cells compared to WT animals. This effect was limited to the early stage of Abeta pathology and was not seen in the 10- or 13-month-old animals. These proliferating DCX^+^ cells were mainly located in the SGCL and had small horizontal processes representing an immature neuroblast morphology (*insert with arrow*). **d** Analysis of Nestin^+^ stem cells and PCNA^+^/Nestin^+^ stem cells specifically in 3-month-old animals showed no changes in the Nestin^+^ stem cell pool of APP-PS1 animals compared to WT. **e** Analysing the Tg2576 mouse model at early stages of pathology confirmed the findings in the APP-PS1 mouse model. At 3 months of age, the Tg2576 mice showed significantly increased numbers of PCNA^+^ cells and significantly increased numbers of PCNA^+^/DCX^+^ cells compared to WT mice. However, these changes were diminished in 5-month-old Tg2576 animals. The number of DCX^+^ cells was not changed in 3- and 5-month-old Tg2576 compared to age-matched WT animals. NeuN was used to stain granular mature neurons and DAPI was used as nucleus stain. Two-way ANOVA with Tukey’s multiple comparisons test was performed (**a**, **b**, **c**, **e**, *n* = 6/group; **d**, *n* = 5/group). *Scale bar* 100 μm (**a**, **b**, **c**, **d**)

As expected, the overall number of PCNA^+^ cells declined with age in WT as well as in APP-PS1 mice, in particular between 3 and 10 months of age (Fig. 2a). This age-related decline in the number of proliferating cells translated in a reduced number of DCX^+^ cells in both genotypes (Fig. 2b). Interestingly, while there was no difference in the proliferation rate between WT and APP-PS1 mice at advanced age, i.e. 10 and 13 months, young (3 months old) APP-PS1 mice showed a significantly higher number of proliferating cells in comparison to WT animals (Fig. 2a). This, however, did not lead to a higher number of DCX positive cells (Fig. 2b). Surprisingly, the numbers of PCNA^+^ cells co-expressing DCX (PCNA^+^/DCX^+^) were significantly increased in 3-month-old APP-PS1 animals compared to WT (Fig. 2c). Most of these proliferating neuroblasts were located in the SGCL and displayed an immature neuroblast morphology (Fig. 2c, arrow). This hyperproliferation of neuroblasts was not detected at later stages, i.e. 10 and 13 months of age, and can be considered as an early event prior to amyloid-beta plaque formation. We further investigated if the hyperproliferation of the DCX cells affected the stem cell pool in the 3 months old APP-PS1 animals by analysing the Nestin^+^ cell population. The number of Nestin^+^ stem cells and the number of PCNA^+^/Nestin^+^ cells was unchanged in 3-month-old AD and WT animals (Fig. 2d), suggesting that the stem cell niche was not depleted as a consequence of hyperproliferation.

The findings of hyperproliferation (PCNA^+^ cells) and hyperproliferating neuroblasts (PCNA^+^/DCX^+^) at very early stages in the APP-PS1 mice were confirmed in the Tg2576 AD mouse model. Here, adult hippocampal neurogenesis was analysed at two time points prior to plaque formation, i.e. in 3- and 5-month-old animals. Very similar to the APP-PS1 mice, the number of PCNA^+^ cells was significantly increased in 3-month-old Tg2576 animals compared to WT, whereas at 5 months of age this effect had already vanished (Fig. 2e). Similar to the APP-PS1 mice, the number of DCX^+^ cells was unchanged in Tg2576 compared to WT animals; however, the number of PCNA^+^/DCX^+^ cells was significantly increased in 3-month-old Tg2576 mice compared to WT. This hyperproliferation of neuroblasts diminished very rapidly and was not detected in 5-month-old Tg2576 animals.

Taken together, we observe early changes in adult hippocampal neurogenesis by terms of increased proliferation and increased numbers of proliferating neuroblasts prior to amyloid-beta plaque formation in two different mouse models for AD.

### Reduced Cell Survival and Decreased Numbers of Newly Formed Neurons in 3-Month-Old APP-PS1 Animals

**Fig. 3:**
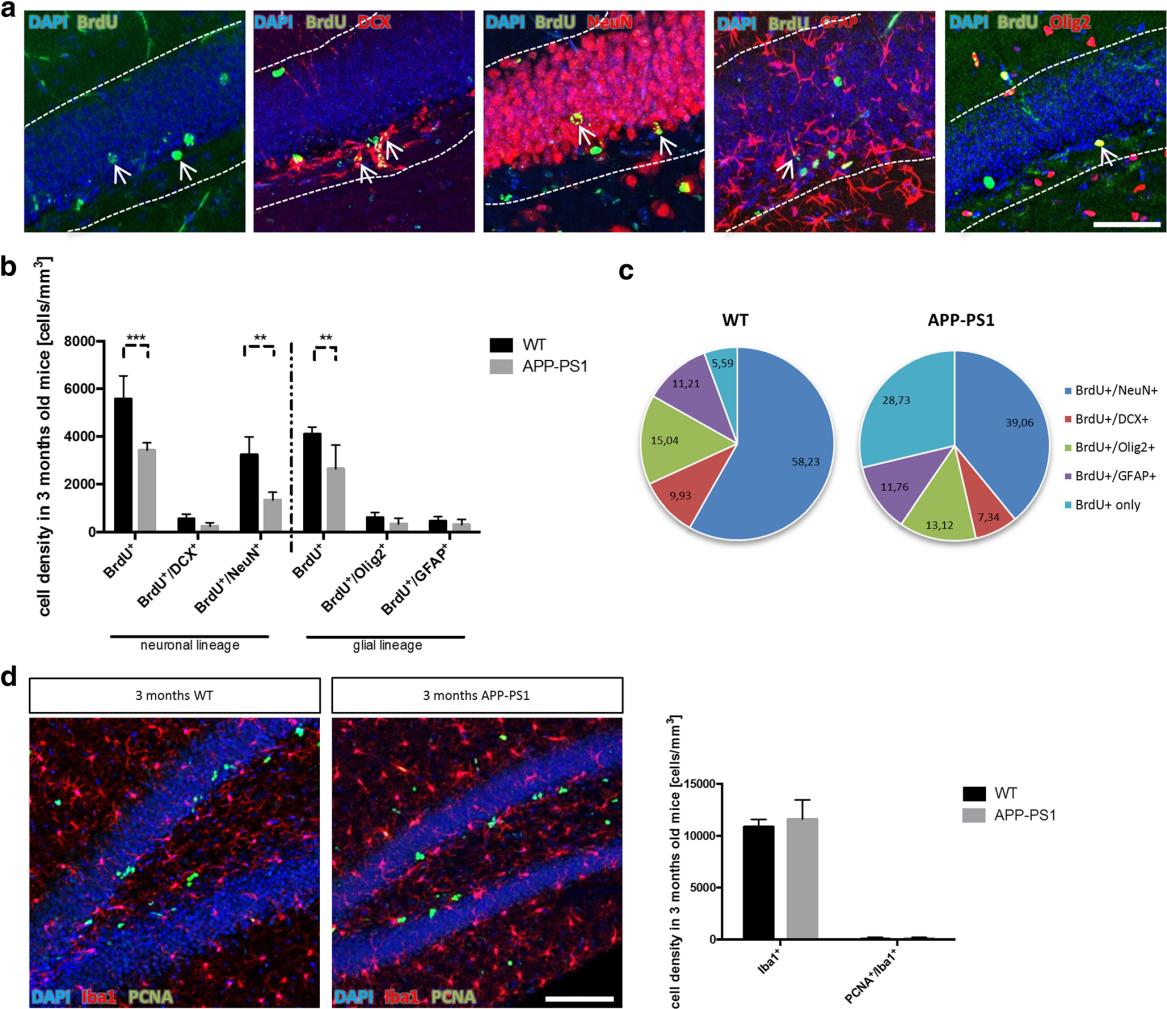
Cell survival and cell fate analysis in the hippocampal neurogenic niche of 3-month-old APP-PS1 mice 30 days after BrdU injection revealed decreased numbers of newly formed neurons. **a** Representative immunohistochemistry images of cell survival and fate analysis showing cell survival (BrdU^+^), newly formed neuronal progenitors (BrdU^+^/DCX^+^), neurons (BrdU^+^/NeuN^+^), astrocytes (BrdU^+^/GFAP^+^) and oligodendrocytes (BrdU^+^/Olig2^+^) in the GCL and SGCL of the dentate gyrus. **b** Quantification of cell survival revealed significantly decreased numbers of BrdU^+^ cells and analysis of newly formed neurons showed specifically decreased numbers of BrdU^+^/NeuN^+^ cells in 3-month-old APP-PS1 animals compared to WT. No changes were observed in the numbers of BrdU^+^/DCX^+^, BrdU^+^/Olig2^+^ or BrdU^+^/GFAP^+^ cells. **c** The percentage of BrdU^+^ cells that differentiated into new neurons (BrdU^+^/NeuN^+^) was clearly reduced in 3-month-old APP-PS1 animals compared to WT (each pie represents 100 % of BrdU^+^ cells). Interestingly, the percentage of BrdU^+^ cells that did not co-localize with any of the neuronal or glial marker examined (BrdU^+^ only) was increased in the APP-PS1 animals compared to WT. **d** The number of microglia (Iba1^+^) and the number of proliferating microglia (PCNA^+^/Iba1^+^) did not change in 3-month-old APP-PS1 animals compared to WT. *White lines* indicate GCL and SGCL of the dentate gyrus. Two-way ANOVA with Tukey’s multiple comparisons test was performed for the neuronal and the glial lineage (**b**, *n* = 4/group) and for microglia (**d**, *n* = 5/group). *Scale bars* 50 μm (**a**), 100 μm (**d**)

Next, we determined if the prominent hyperproliferation of DCX^+^ cells observed at the 3-months pre-plaque age translates into changes in cell survival and differentiation fate. Therefore, we analysed the survival rate and cell fate of the newly formed cells in the hippocampus of 3-month-old APP-PS1 mice. Animals received BrdU at 5 consecutive days starting 30 days before perfusion, and survival and fate of proliferating cells (BrdU^+^ cells) in the DG was analysed by co-staining with markers for neuronal progenitors (BrdU^+^/DCX^+^), newly formed neurons (BrdU^+^/NeuN^+^), astrocytes (BrdU^+^/GFAP^+^) and oligodendrocytes (BrdU^+^/Olig2^+^) (Fig. 3a).

Interestingly, in contrast to the hyperproliferation, the number of BrdU^+^ newly formed cells surviving the period of 30 days was significantly reduced in APP-PS1 compared to WT mice (Fig. 3b). Analysis of cells committed to the neuronal lineage (BrdU^+^/DCX^+^; BrdU^+^/NeuN^+^) revealed that after 30 days of BrdU injection, most of these previously dividing cells were already differentiated into NeuN^+^ new neurons (WT = 58,23 %; APP-PS1 = 39.06 %). The number of BrdU^+^/DCX^+^ cells was very low (WT = 9.93 %; APP-PS1 = 7.34 %), and did not differ significantly between APP-PS1 and WT animals. In contrast, the number of newly formed neurons (BrdU^+^/NeuN^+^ cells) in APP-PS1 animals was significantly reduced compared to WT (Fig. 3b, c). Analysis of the glial lineage did not show any changes in the number of BrdU^+^/Olig2^+^ or BrdU^+^/GFAP^+^ cells between APP-PS1 and WT animals (Fig. 3b, c). Interestingly, when analysing the potential fate of the BrdU^+^ cells, we noticed that APP-PS1 mice showed a decreased percentage of BrdU^+^/NeuN^+^ cells and a bigger pool of BrdU^+^ cells (BrdU^+^ only) that did not co-localize with any of the neuronal or glial lineage markers analysed (Fig. 3c).

Another cell type showing a considerable amount of proliferation in the adult brain, in particular under pathological conditions, is microglia. To test if microglia contribute to the observed hyperproliferation in 3-month-old APP-PS1 mice, we quantified the number of microglia (Iba1^+^) and proliferating microglia (PCNA^+^/Iba1^+^) in the GCL and SGCL of the dentate gyrus in 3-month-old APP-PS1 and WT animals. The numbers of Iba1^+^ cells and PCNA^+^/Iba1^+^ cells, the latter being extremely low, were not different in 3-month-old APP-PS1 compared to WT animals (Fig. 3d), suggesting that the observed increase in PCNA^+^ proliferating cells in 3-month-old APP-PS1 mice was not due to increased microglia proliferation.

Summarizing these observations, we conclude that in the hippocampal neurogenic niche of 3-month-old APP-PS1 mice, even though amyloid-beta plaques are not yet present, the survival of proliferating cells and their neuronal differentiation fate is significantly reduced in APP-PS1 compared to WT mice.

## Discussion

The present work demonstrates early alterations in hippocampal neurogenesis in two different amyloidogenic animal models of AD, the APP Swedish PS1 dE9 and the Tg2576 mice. Surprisingly, even before the appearance of any visible amyloid-beta plaques, neurogenesis was already affected. We noticed a strong hyperproliferation of DCX expressing neuroblasts specifically at the pre-plaque stage of pathogenesis. This, however, did not translate into a net increase in neurogenesis, as the survival of the newly generated cells was compromised already at this stage of pathogenesis. Even though this is purely speculative at this point, a surplus of hyperproliferating neuroblasts in the dentate gyrus might have functional consequences for the hippocampal network. It is well established that immature neurons in the adult hippocampus are electro-physiologically active, they are highly excitable and modulate the activity of the hippocampal circuitry [reviewed in 41–43]. Moreover, they are required for learning as their depletion in an experimental animal model abolishes the successful acquisition of spatial and reversal learning [27]. One might speculate that the surplus of young proliferating immature neurons in the hippocampus at this pre-plaque stage might relate to well-known alterations of hippocampus function in prodromal AD in humans such as hippocampal overactivity as determined by functional MRI [44, 45]. An overactivity of the hippocampus at early stages of AD resulting in amnestic mild cognitive impairment (aMCI), is a dysfunctional condition being responsible for episodic memory loss in patients [46–48]. Treatment of aMCI patients with the anti-epileptic drug levetiracetam reduced the hippocampal hyperactivity to levels of healthy controls and even improved their memory function in memory tasks [49]. It is very likely that hyperactivity of the hippocampus in prodromal AD stages is consolidated or even provoked by changes in neurogenesis such as increased proliferation of DCX expressing neuroblasts in the dentate gyrus. Alternatively, the hyperproliferation of DCX cells might relate to the increased seizure activity early in human AD and in AD mouse models [50, 51]. Video EEG recording of 3- to 4-month-old APP Swedish PS1 dE9 mice revealed 65 % of AD animals developing at least one electrographic seizure, with higher incidence at more advanced age [52]. Either the hyperproliferation of neuroblasts might change hippocampal circuitry and contribute to seizures, or vice versa, the seizures might induce a hyperproliferation of the DCX cells. Indeed, seizures are well known to increase hippocampal progenitor proliferation and the number of DCX expressing neuroblasts [53, 54, reviewed in 55].

The present study complements a number of previous studies that had analysed adult hippocampal neurogenesis in different mouse models for AD, however with controversial results [reviewed in 19]. Conclusive evidence for decreased or increased neurogenesis in AD mouse models is not easily possible, taking into account the different ages of analysed mice cohorts studied, their individual genotypes corresponding to the mouse model, and the specific analysed cell populations in the different neurogenic niches. Nevertheless, the majority of studies report about decreased neurogenesis in AD mouse models [reviewed in 34]. In the APP Swedish PS1 dE9 animals, reports were mainly on animals with already settled amyloid-beta plaque formation and these studies mostly concluded a reduction in neurogenesis. Taniuchi et al. describe lower cell proliferation, i.e. reduced BrdU^+^ cell numbers after 3 days of consecutive BrdU injection, and reduced numbers of neuroblasts (DCX^+^ cells) in 9-month-old APP Swedish PS1 dE9 animals [32], i.e. the same model as in the present study. Additionally Taniuchi et al. analysed 5-month-old APP-PS1 animals; however, they did not observe any changes in neurogenesis at this age. Niidome et al. showed decreased numbers of proliferating cells (BrdU^+^ cells) in the SGCL of 9-month-old APP Swedish PS1 dE9 mice [58]. In contrast, the present analysis did not reveal changes in dentate gyrus cell proliferation or in the number of neuroblasts in 10-month-old APP Swedish PS1 dE9 animals, i.e. a stage with severe plaque pathology. This might be due to the different methods used to detect cell proliferation. We analysed proliferation by the cellular expression of PCNA, a protein expressed by proliferating cells in late G1 and S-phase of mitosis [59]. Others used BrdU labelling as a proliferation marker, which, depending on the time between cell labelling and animal perfusion, can provide different results. At the level of survival and fate of the newly generated cells, Verret et al. report reduced cell survival, i.e. the number of BrdU^+^ cells that were labelled 4 weeks before, and reduced numbers of newly formed neurons (BrdU^+^/NeuN^+^ cells) in the hippocampal dentate gyrus of 6-month-old double transgenic APP Swedish PS1 dE9 mice, which at this stage have already prominent amyloid-beta plaque depositions in the hippocampus [60]. We demonstrate that the reduction of cell survival and formation of new neurons is evident already early in the development of Abeta pathology, prior to amyloid-beta plaque formation. This is consistent with the work by Demars et al., which describes reduced neuronal differentiation in 2-month-old APP Swedish PS1 dE9 animals [61].

The changes in neurogenesis at the pre-plaque stage were not restricted to the APP Swedish PS1 dE9 animals but also evident in the slower disease progression Tg2576 model. This is in agreement with the work by Krezymon et al., which describes increased numbers of proliferating cells in the hippocampus of 3-month-old Tg2576 mice [33]. Furthermore, Krezymon et al. showed decreased numbers of newly formed neurons (BrdU^+^/NeuN^+^ cells) at this very early stage in the Tg2576 mouse model. The newly formed neurons had shorter and less arborized dendrites, fewer dendritic spines, and shorter axons and were not integrated into the hippocampal circuitry ultimately leading to a reduced survival of these cells [33].

What triggers the early changes in neurogenesis? Obviously, amyloid-beta forms might induce changes in the activity of hippocampal neural stem- and progenitor cells or even in the newly generated neurons. Soluble amyloid-beta peptides at low concentrations have a trophic effect and promote the survival of neurons [62]. Also, other proteolytic products from APP, e.g. the sAPPalpha product, stimulate proliferation of adult SVZ NPCs [63]. The effects of amyloid-beta and its different forms on neural stem cells (NSCs) are still controversial [reviewed in 21, 64]. For example, only aggregated amyloid-beta_1–42_ was able to induce neurogenesis in vitro. Thereby it is not affecting proliferation of murine NSCs; however, it increased the number of total neurons in vitro. Interestingly, only aggregated amyloid-beta_1–42_ and oligomeric amyloid-beta forms were able to increase neurogenesis in vitro, whereas non-aggregated forms of amyloid-beta_1–40_ or amyloid-beta_1–42_ had no effect on the NSC cultures [65]. Another report demonstrates that amyloid-beta oligomers reduce proliferation of human neural stem cells and alter the fate of the newly generated cells from neuronal towards the glial lineage [66]. Fibrillary amyloid-beta forms are heavily discussed to reduce neurogenesis in vitro. Human embryonic stem cell cultures and respective neurosphere cultures either treated with fibrillary amyloid-beta or conditioned media of human microglia treated with fibrillary amyloid-beta decreased neuronal differentiation and switched differentiation towards glial cells in vitro [67]. In vivo, human amyloid-beta_1–42_ injected into the hippocampus of adult mice inhibited neurogenesis [68]. Thus, the effect of amyloid-beta on neurogenesis is still controversial [reviewed in 7].

The increase in the number of proliferating neuroblasts is not limited to the prodromal stages of AD pathology but is also seen in other neurodegenerative diseases suggesting that other non-APP specific factors might contribute to modulation of neurogenesis in neurodegeneration. In rat models for Huntington disease, an increased number of proliferating neuroblasts can be detected at early stages of disease as well [69]. Also, in alpha-synuclein transgenic models for Parkinson’s disease (PD), similar findings of reduced cell survival and reduced new neuron numbers are reported in 4-month-old mice, before PD symptoms are observed [70]. A mechanism common to the various neurodegenerative diseases modulating neurogenesis might be neuroinflammation, in particular microglia activity [reviewed in 71]. We addressed this issue in the present work, but did not see any changes in the number of microglia or in the number of proliferating microglia.

Prodromal changes in neurogenesis are described in a variety of neurodegenerative diseases. Therefore, adult hippocampal neurogenesis is a process that is highly sensitive to changes and is already reflecting very early changes to the brains microenvironment. Our findings suggest that first changes of the neurogenic niche environment occur at very early prodromal stages of Abeta pathology and these alterations already have an impact on cell proliferation, cell survival and cell fate. Further investigation in particular on the functional relevance of the hyperproliferating DCX cells will be required.
